# Automatic Construction of 3D Basic-Semantic Models of Inhabited Interiors Using Laser Scanners and RFID Sensors

**DOI:** 10.3390/s120505705

**Published:** 2012-05-03

**Authors:** Enrique Valero, Antonio Adan, Carlos Cerrada

**Affiliations:** 1 School of Computer Engineering, Universidad Nacional de Educación a Distancia (UNED), C/Juan del Rosal, 16. 28040 Madrid, Spain; E-Mail: ccerrada@issi.uned.es; 2 3D Visual Computing and Robotics Lab, Universidad de Castilla-La Mancha (UCLM), Paseo de la Universidad, 4. 13071 Ciudad Real, Spain; E-Mail: Antonio.Adan@uclm.es

**Keywords:** 3D modeling, RFID, laser scanner, 3D data processing

## Abstract

This paper is focused on the automatic construction of 3D basic-semantic models of inhabited interiors using laser scanners with the help of RFID technologies. This is an innovative approach, in whose field scarce publications exist. The general strategy consists of carrying out a selective and sequential segmentation from the cloud of points by means of different algorithms which depend on the information that the RFID tags provide. The identification of basic elements of the scene, such as walls, floor, ceiling, windows, doors, tables, chairs and cabinets, and the positioning of their corresponding models can then be calculated. The fusion of both technologies thus allows a simplified 3D semantic indoor model to be obtained. This method has been tested in real scenes under difficult clutter and occlusion conditions, and has yielded promising results.

## 1. Introduction

The concept of “intelligent environment” arose in the late 90s to characterize a set of technological elements which were capable of communicating with the user and making decisions by means of an intelligent process. This interaction between user and device is carried out in a transparent manner, in the sense that the presence of these elements is sufficient to establish communication between them. In [1], the authors afford a solution to link contextual information with user interactions from daily activities, in an implicit and transparent way.

One of the best known examples concerning intelligent environments is radio frequency identification (RFID) technology. RFID systems are able to store and retrieve significant data from identified items by means of small tags in which this information can be easily read and written. These small tags can be attached to or incorporated into products, animals or people, and contain relevant information about the associated item, thus permitting their identification and the control of some of their features.

The relative low cost of this technology, together with its rapid development and the tags' adaptability for use on a great variety of surfaces have led to the constant expansion of RFID technology over the last few years. There are diverse fields of application of this technology. In the fields of manufacturing and distribution, RFID systems have been used in the identification of packing and containers and for component tracking in factories [2,3]. Moreover, savings of time and paper are achieved in these cases because the inspection registers can be certified by writing on the tags which are attached to the elements [4]. Other authors [5] propose an application for a conference site through identification by RFID. In this work, people attending the conference wear RFID tags and the session room is equipped with antennas which detect the presence of the speakers in the room and facilitate the proceedings. This intelligent technology is also used in medical environments to locate the hospital equipment and improve its management, control patients' activities in hospitals, or help people who need assistance [6].

RFID technology has also been applied in the field of robotics where it has been used as a complementary technology to laser sensors for robot localization and for environmental map construction, for example in [7]. The robotic platform described in [8] has been built to assist the visually impaired in supermarkets or airports. The intelligent system helps them to avoid collisions or to indicate how to follow itineraries. Tong and Zekavat [9] propose the use of tags and readers for the safer navigation of mobile robots, by avoiding collisions and estimating times and directions of arrival. Other authors [10] combine RFID technology with GPS to improve the precision of the navigation systems in road vehicles. RFID and 3D scanners has been already used by other authors [11] in computer vision field with recognition purposes. They detect the presence of a given object in a scene by reading the RFID-EPC code of the tag attached to it. Presence information helps to alleviate the searching processes, achieving significant computation time reduction in the object recognition algorithms. However, in this work only an identification code for every object is written in the tags but information concerning to dimensions, type of element or color is not provided.

## 2. RFID and Laser Scanning Technologies in Buildings

### 2.1. RFID Applications

Our work with RFID technologies is framed in the area of construction, particularly in civil and industrial buildings. RFID technology fits well into the Building Information Modeling (BIM) standard that won general acceptance in the Architecture Engineering and Construction (AEC)-field several years ago. The advantage of BIM is that the entire life-cycle of the building is covered by a unified numerical model. RFID tags help to identify elements of this model on-site. A wide number of references exist in the AEC field. RFID technology is becoming a very useful tool in this research field because of its rapidity in identification and information localization, together with the tags' durability and the high range of action of these systems.

Linking the RFID technology to the construction industry is a challenge which began the late 90s, particularly in areas such as vehicle access control, personnel control, tool maintenance, or cycle times and resource tracking. In fact, the principal application of the RFID in the construction processes is currently the tracking of materials. Several authors [12,13] report endowing materials with tags in order to know their movements within the area of construction from reception to installation. Other authors propose the fusion of technologies by equipping the tag readers with GPS antennas to obtain the precise position of the constructive elements [14,15], or by verifying the progress of the activities by means of laser sensors and photogrammetric techniques in addition to RFID [16]. Works linked to the study of the traceability of pipe spools also exist [4,17,18]. An interesting application presented in [19] refers to the prevention of accidents caused by collisions in places under construction. Structures, machinery and workers are provided with tags and readers that allow their risk in the scenarios to be evaluated. This technology is also used for the management and control of workers' punctuality [4,20], and the access control of vehicles to the construction area [21]. Numerous works can also be found in the field of maintenance and inspection. Kondo *et al.* [22] present a study on the draining of a system of pipelines. They use a set of tagged balls both to automatically verify the evacuation time of each one and to check the correct operation of the system.

Information management in the areas of civil engineering and construction goes beyond the constructive process. It is also necessary to record certain data during the lifespan of the building. The safety of a building can be improved, for example, with a good management of its set of fire-extinguishers, by indicating on their tags whether any inspection is required, whether they work properly or where they are located [23]. Moreover, the application proposed in [24] may assist rescue teams in a fire. Information from different tags relevant to a building's fire control (maps, number of plants, location) is gathered and sent by means of a PDA system with RFID. With regard to maintenance and inspection, Cheng [25] proposes an information system for the maintenance of open buildings. The tracking of the elements comprising the building that must be evaluated for their replacement is carried out with this application.

### 2.2. Laser Scanning Applications

Our work is focused on the automatic construction of 3D basic semantic models of inhabited interiors using laser scanners with the help of RFID technologies. An inhabited environment involves certain disorder in the scene: there are objects on tables, in racks, papers stuck to walls and to windows, *etc*. Moreover, some elements in a scene may occlude others, signifying that they cannot be entirely sensed by a laser scanner.

In the field of laser scanning applied to buildings, there are some lines of work particularly developed during the last years. Different techniques for the automatic reconstruction of as-built facilities are presented in [26]. The authors review several previous works focused on basic structures recognition, geometric modeling and constructive elements relationship.

As regards the detection and modeling of single objects or parts of large scenarios, Kwon *et al.* [27] introduce a set of algorithms which fits sparse point clouds to single volumetric primitives (cuboids, cylinders and spheres). The algorithm is extended to groups of primitives belonging to the same object. Another work [28] identifies and localizes relevant kitchen objects including cupboards, kitchen appliances, and tables. They interpret the point clouds in terms of rectangular planes and 3D geometric shapes. Valero *et al.* [29] focus on the modeling of those linear moldings that typically surround doorways, windows, and divide ceilings from walls and walls from floors.

In [30] an automated recognition/retrieval approach for 3D CAD objects in construction context is presented. Bosche [31] proposes a semiautomatic method to match 3D existing models to the collected data in industrial building steel structures. The author develops a variant of the ICP algorithm to recognize CAD models objects in large site laser scans. The same author presents in [32] a plane-base registration system to coarsely align laser scanners data with the project 3D models in the AEC/FM industry context.

As regards walls and façades detection, detailed models of part of these components are obtained in [33–36]. Bohm *et al.* [33,34] propose a method where the data processing goes from detecting windows through low data density regions to discover other data patterns in the façade. Important façade elements such as walls and roofs are distinguished as features in [35]. Thrun *et al.* developed a plane extraction method based on the expectation-maximization algorithm [37]. Other researchers [38,39] have proposed plane sweep approaches to find planar regions. Früh *et al.* [36] develop a set of data processing algorithms for generating textured façade meshes of cities from a series of vertical 2D surface scans.

To perform the automatic reconstruction of a scene, we process a dense cloud of points to identify certain objects whose 3D models are stored in a knowledge base. Owing to the occlusions and the complexity of the geometry of the scanned elements, the identification of objects from the cloud of points generated by the scanner is a very difficult task. To date, few papers have tackled the occlusion problem by using range data for indoor reconstruction. Papers which process laser scanner data to model predominantly planar surfaces, such as walls, floors, and ceilings, can be found in [40]. In [41], the authors are also able to detect and model openings, and fill in the occluded regions. Of course, many more related papers exist which use range images and deal with specific topics such as plane detection, wall identification, 3D scene segmentation, the reconstruction of surfaces, detailed, precise, momentary occlusion and hole-filling, but none of them provide an extended solution in complex scenes covering all those aspects. Adan [42] presents a method to automatically convert the 3D point data from a laser scanner positioned at multiple locations throughout a building into a compact, semantically rich model. In this work, the main structural components of an indoor environment (walls, floors, ceilings, windows and doorways) are identified despite the presence of clutter and occlusion.

As has already been mentioned, this article proposes a solution based on the combination of two technologies of a different nature, these being laser scanners and radio frequency identification. RFID tags adhered to different objects provide valuable information about them, which makes the identification and positioning of basic elements in the scene faster and easier. In this work, we propose an innovative approach in this challenging research field. Obtaining an accurate model of an interior can be very useful in applications related with exploration, manipulation and autonomous navigation with mobile robots or vehicles. In these cases the robot needs to know the principal constructive elements of the interior, the objects in the scene and their position.

The paper is organized as follows: Section 3 presents the structure of the proposed system, based on two different sensorial technologies. In Section 4 we show the information that is available from both the laser scanner and the RFID sensor. Section 5 proposes a solution by which to identify and calculate the pose of basic elements (both static and dynamic) in a facility. Finally, Section 6 shows the experimental results obtained with our proposal.

## 3. Overview of the System

**Figure 1.**
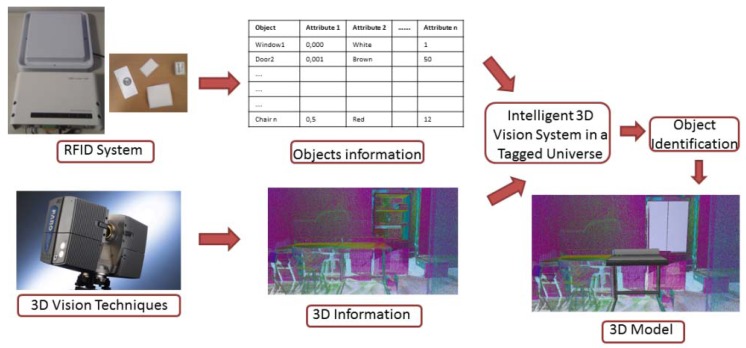
Overview of the system proposed in this work, where 3D vision and RFID technologies are fused.

The outline of the proposed system is depicted in Figure 1. The system is based on two technologies connected by a specific integration block. The treatment of 3D information in the scene, represented by the 3D Vision Techniques block, considers both the acquisition and the processing of 3D data. A FARO Photon 80 scanner has been used in this block, and the data obtained from this 3D sensor provides geometric and color information of the scene. Bearing an automatic generation of the model of the scanned scene in mind, it is necessary to recognize the elements that are present in the scanned zone. RFID technology can assists vision technologies in this task by providing relevant information to identify and pose the elements in the scene. The RFID System block represents the treatment of the information stored in the passive RFID tags. An OBID sensor, model LRU 3500 from FEIG Company, connected to an ultra-high frequency (UHF) antenna has been used in this block. This sensor allows information to be read and written on the tags. Both, 3D scanner and RFID system, work together during the scanning process. The RFID reader is onboard the scanner platform. Thus 3D data and tag detection can be obtained at the same time. We denote this system in Figure 1 as “Intelligent 3D Vision System in a Tagged Universe”, in which data from the two sensors is used to identify elements and to reconstruct the 3D model of the scenario.

**Figure 2.**
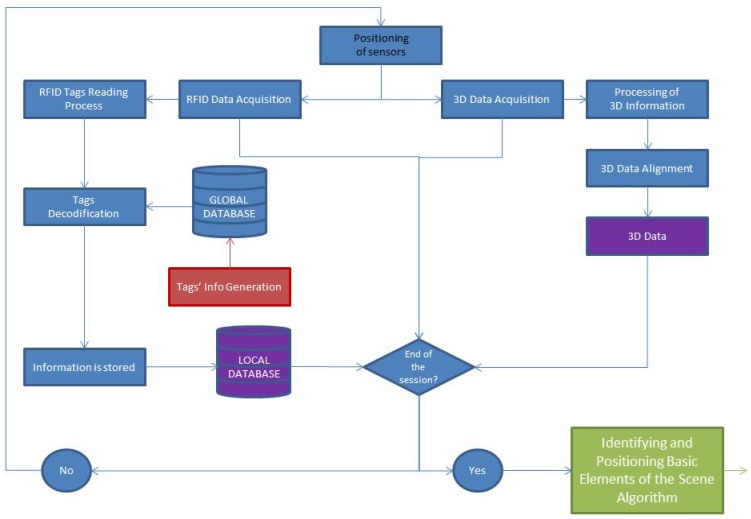
Flowchart of the acquisition data process.

With the purpose of obtaining a general view of the process carried out in this work, a flowchart is presented in Figure 2. This diagram shows the different methods executed during the data acquisition by means of the above mentioned sensors.

At first, the two sensors (scanner and RFID reader) are placed together in several strategic locations of the scene to cover the whole indoor environment. For each of these positions, a data acquisition process is carried out. A set of 3D points is provided by the laser scanner and at the same time, certain geometry and color information of the different elements is obtained from the tags by means of the RFID sensor.

The 3D point clouds, which are acquired from different positions of the inhabited interior, are pre-processed and registered in accordance with a universal coordinate system (UCS). These two datasets, corresponding to 3D points and object information (in purple in Figure 2), are the inputs of the algorithms which identify and pose the basic elements in the scene. These algorithms will be explained in Section 5.

## 4. Available Information from Laser Scanner and RFID

### 4.1. Laser Scanner

**Figure 3.**
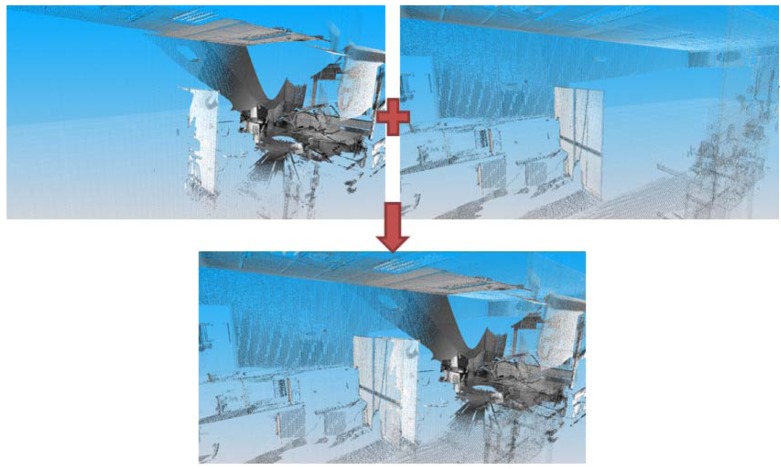
Joint registering of two digitalizations of a scanned inhabited interior.

3D information of the environment is obtained by virtualizing the scenario by means of the laser scanner sensor mentioned in the previous section. Its field of vision allows it to be employed in scenes under study up to 80 m. The information provided by the sensor is associated with the geometry and the texture of the scanned room. Several acquisitions of the same room are carried out from different points of view. Then, all the range images provided by the sensor are filtered and transformed to a universal coordinate system in a first pre-processing phase. For a given acquisition, several fiducials are identified in a planar image of the scene and the user marks their centers of mass. A set of correspondences between the center points from two different sensor positions are manually established, and a transformation matrix is obtained between both reference systems by means of commercial scanning software. The same procedure is followed with the next sensor positions, having considered the first sensor position as the universal coordinate system. All the data thus remain registered in a common reference system, as do all the sensor positions considered. This registering process is habitual in the field of the digitalization of interiors or exteriors in buildings. Figure 3 shows the registration of two digitalizations of an inhabited interior.

All the available geometric information of the scene consists of an unconnected set of points which is gathered after registering all the 3D data under a common reference system. Certain geometric features can be extracted from the treatment of these data which allow the automatic segmentation and recognition of certain components. Walls, ceilings, floors, doorways and windows or the frames around them can be obtained in this manner. Nevertheless, these recognition processes are extremely complex in inhabited interiors.

### 4.2. RFID

RFID technology is an information exchange system for automatic identification inspired by radio frequency waves. The information is stored in devices named tags or transponders. Three types of tags exist: active, passive and hybrid. Active tags incorporate the power supply for their circuits and propagate the signal to the reader. Passive tags obtain the required energy by induction from the readers. Passive tags can be used for distances of up to approximately 15 m, whereas active tags have a much bigger range of action (up to 500 m) and can store a large quantity of information. Hybrid tags can transmit, but they have to be told to transmit. They need to be turned on by a signal, as could be a satellite.

The system's range of action is also influenced by the type of antenna installed in the reader. The use of a low frequency (LF) antenna or a high frequency (HF) one thus allows operating distances in the range of dozens of centimeters, whereas the installation of an ultra-high frequency (UHF) antenna allows communication in the range of meters.

The previous information will allow the reader to deduce that the greatest range of communication is achieved by the use of UHF antennas and active tags. In our particular case, where the required working range is of dozens of meters and where it is necessary to modify the tags' content, we have used UHF antennas (865–868 MHz) and passive tags with a storage capacity of up to 512 bytes.

**Figure 4.**
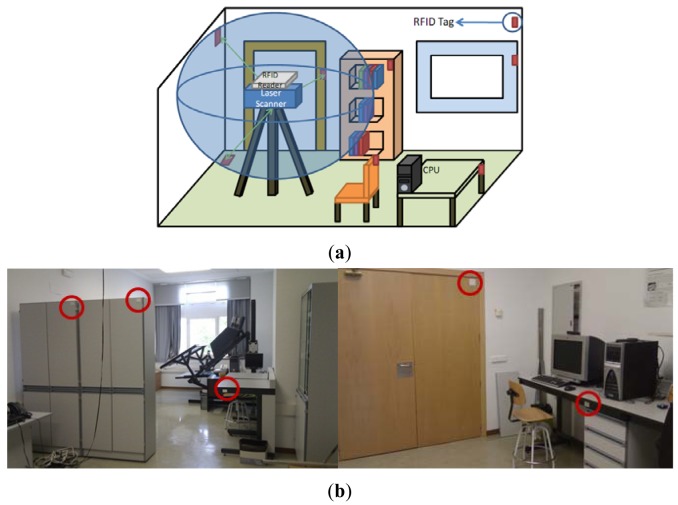
(**a**) Diagram representing the laser scanner and the RFID system in a room. (**b**) Several tags placed on dynamic elements of the room.

Figure 4 shows that the tags are located in a disperse manner in the scene and, as has been mentioned previously, they contain information about attributes corresponding to the objects to which they are adhered. One advantage of this technology as opposed to others such as bar codes or infrared light is that the tags do not need to have a direct vision of the reader to gather the information. They can therefore be attached to any of the surfaces of the objects located in the scene. Nevertheless, if the tag is attached over a metallic surface, it must be placed approximately 1 cm away from the surface to be properly detected.

In our work, tagged elements are classified into static (structural) and “dynamic” (equipment). Until now, the model of the scene has been reduced to a set of static and dynamic basic elements. The essence of our research is to obtain the complete model of the scene. We define some objects as “dynamic” because they can be moved or even removed in the same scenario, depending on the time in which the scenario is sensed.

The objects considered and the information that is stored for each one are shown in Table 1. Static elements concern those which can be found in an empty room before it is furnished. They are basically: walls, openings, moldings and columns. In the following paragraphs we present the information contained in each particular RFID tag.

- Wall tag. This contains the properties of the wall at the moment at which the scene is scanned: wall type (ceiling, floor, wall), wall dimension (length, width), paint color, number of openings on the wall.
- Opening tag. This concerns the holes in the walls which are usually covered by other structures. An opening is characterized by: type (door, window, closet and pure hole), opening dimension ((length, width) and color.
- Molding tag. It may be a molding on the opening or a molding on the wall. The information is as follows: type (wall molding and opening molding), molding dimension (length) and color.
- Column tag. The columns correspond to the pillars which can be found in the scene but not next to the walls. We distinguish between cylinders and square pillars.

The so called “dynamic” elements, are those which can be added, removed or changed with regard to its initial position in the scene. The approach presented in this paper considers the minimum number of dynamic elements which can be found in an inhabited scene. To date we have distinguished three dynamic elements: tables, chairs and wardrobe/filling cabinet. There are a wide variety of shapes for each type of dynamic element. In order to facilitate the matching process between the model corresponding to the tag recognized by the RFID and the 3D data provided by the laser scanner, we include basic information of the models. The main idea is to carry out a model based segmentation of the 3D data in order to carry out a further model-data matching process.

For example, for table tags we include only the information which is related to the table top (area and height) so that we can search for 3D data fitted to a plane for a particular distance from the floor. A description of dynamic tags is as follows:

- Table tag. Any type of table is characterized solely by the height and area of the top. It does not make sense to store more detailed information because, owing to the occlusion and the incomplete scanning of the scene, information concerning a part of the table's surface might not be available. Figure 4 shows an example in which part of the table is missing.
- Chair tag. Here we distinguish between simple chair and sofa elements. Chair tags only contain information concerning the chair's legs (length and separation) whereas sofa tags store information about surface and volume.
- Wardrobe/filling cabinet tags. These elements tend to be parallelepipeds and the scanner usually provides vertical or horizontal flat faces. We therefore store the dimensions of the element's three planes.

Although color information is also stored in all the aforementioned tags, in this paper it is not used as discriminatory feature. This is owing to the fact that color might be a highly variable property which strongly depends on the illumination of the scene. This challenging issue will be tackled in the future.

Note that tags coordinates in the world reference system are not necessary in this work since we only need to identify the object in which the tag is placed.

## 5. Identifying and Positioning Basic Elements of the Scene

**Figure 5.**
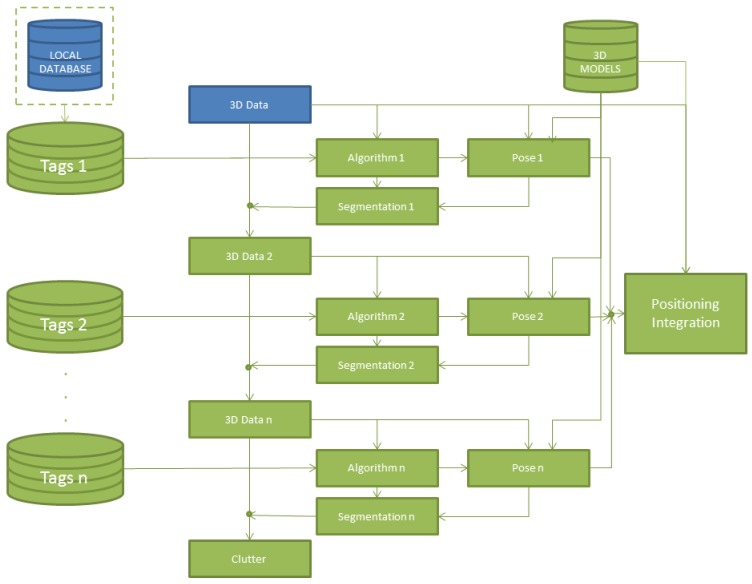
Flowchart of the data segmentation process.

The general strategy is presented in the diagram of Figure 5.

It consists of carrying out a selective and sequential identification of the elements of the scene. Each element is segmented from the cloud of points by means of a different algorithm depending on the information that the RFID tag provides. Whenever an element is identified, its pose is calculated and its corresponding 3D points are removed from the cloud of points before searching for the following element. Once the pose is calculated for each element, the corresponding 3D model is integrated in a virtual scenario. The identification sequence for the elements of an inhabited interior is the following: static elements (walls, ceiling, floor, openings in the walls and moldings) and dynamic elements (tables, closets and chairs).

### 5.1. Static Elements

After registering 3D data from the different positions of the laser scanner, we can recover all available geometric information from the scene: a multitude of unconnected 3D points must then be processed. As will be shown later, apart from outliers and noise, the point density is usually irregular owing to overcrowded and low density areas on the walls. The volume of the interior room is obtained through the walls' positions and 3D data corresponding to other rooms or outside space are removed. Therefore, determining the volume of the room does not mean giving a quantity in cubic meters in advance but establishing the boundaries of the room after determining the position of the walls.

The projection of the 3D data from a specific viewpoint allows us to obtain a normalized binary image in which each pixel can be occupied by one or more 3D points (white pixels in Figure 3(b)) or not. From here on, we will denote by *I* the projected image of the data from a top view. This image will help us to obtain a coarse location and position of the walls which will be later refined. The segmentation process is as follows.

After creating image *I*, the boundary of the room is extracted and, through a Hough Transform algorithm, the set of edges corresponding to the walls in the projected image are detected in a 2D context. Afterwards, we figure out the intersections between edges and obtain the corners in the image. Assuming vertical walls, the segments in the image generate planes and the corners, the edges between planes. These planes are used to segment the original points lying into each wall. We then calculate the mean square distance of the point cloud to the walls and classify each point into a wall.

**Figure 6.**
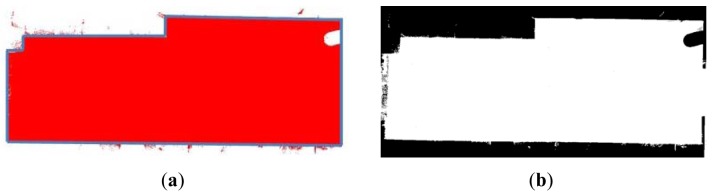

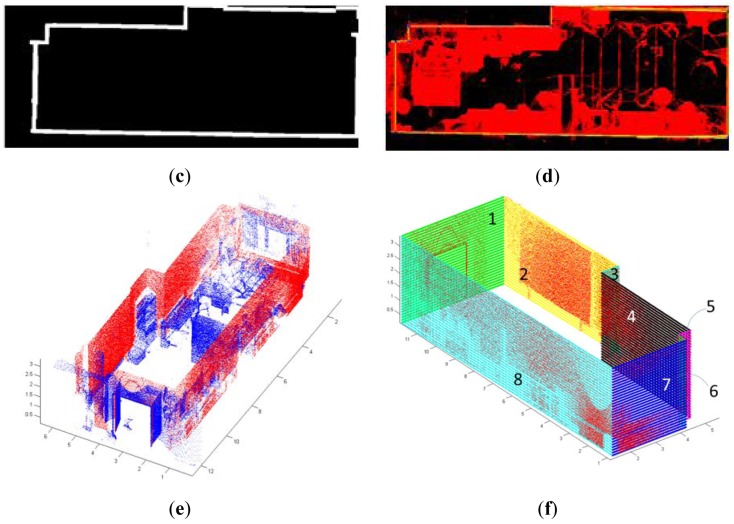
Stages in a wall segmentation process. (**a**) Visualization of the point cloud from a zenital viewpoint. (**b**) Binary image generated after discretization. (**c**) Boundary detection. (**d**) Definition of edges and corners in the image. (**e**) Walls obtained from the point cloud. (**f**) Wall labeling and plane fitting.

Figure 6 shows the steps of the segmentation process: (a) 3D point cloud viewed from the top of the room; (b) Discretization of the view and generation of binary image *I*; (c) Boundary extraction in *I*; (d) Edge and corner detection.

Once the walls' points are identified, the next process consists of labeling the different walls and fitting each one of them to a plane. Thus, we obtain a simplified 3D model of the room. A simple model in this context signifies a model which contains the labels, the pose and the relationship between the objects in the scene. Complex semantic models are those that include much more information like sort of material, history, physical and chemical properties. In Figure 6(e) the points corresponding to the walls are represented in red. In (f) the fitted patches are shown in different colors.

In each plane fitted to a wall we distinguish between three types of regions: empty (A), occupied (B) or occluded (C). This labeling stage is obtained after carrying out a ray-tracing process from each of the laser scanner positions. In order to discover which parts of the wall must be filled and which must not, a segmentation algorithm of wall occluded regions is carried out. Finally, openings (*i.e.*, regions which are not a part of the wall structure, such as doorways and windows) are detected using the learning SVM technique published in [43]. More details about the wall surface extraction can be found in an earlier work in [41]. At this point, the approximated geometry of the room is known. Information from the tags is then used to create a refinement process in which the boundaries of walls, ceiling and floor are accurately fitted and integrated until a polyhedron is attained. The size and position of the openings in the wall are also refined according the data contained in the associated tags. Finally, points corresponding to moldings are detected around the openings and the associated molding models are inserted into the room model.

### 5.2. Dynamic Elements

**Figure 7.**
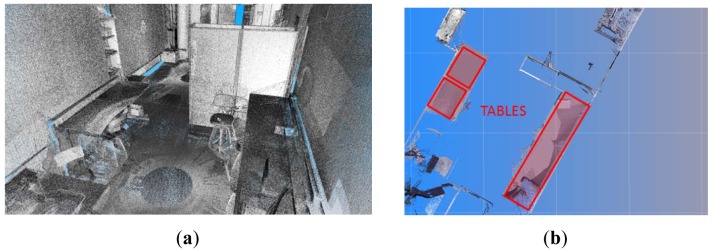
(**a**) Cloud of points including different table models. (**b**) Data slice containing detected table tops.

Once the static elements have been identified and reconstructed, the next step consists of identifying each of the dynamic elements present in the scene. As was mentioned previously, in order to facilitate the identification of tables, information related to the height from the floor and the area of the top is stored in the RFID tags adhered to them. A search of flat surfaces at certain height is carried out when a table tag is read. Once a table is detected, we take one horizontal slice at the height of the table top and process the points which fall into it. In the first step, we project the slice from a top view and generate a 2D image in which the pixels represent the points of the slide. After this, we segment the image and calculate the pixels of maximum curvature. Since sparse areas may appear in the image, a dilation filter is previously applied in order to obtain compact regions in which high curvature points can be discovered in a more robust manner. Harris's method [44] has been used to compute corners in the compact regions. The next step consists of recovering the corresponding 3D coordinates and calculating an approximated pose of the table's model in the scene. By matching the maximum curvature points of the data and the ones of the top table's model, this can be roughly positioned (see Figure 7). The exact position of the table's model in the scene is computed by means of an ICP algorithm [45].

**Figure 8.**
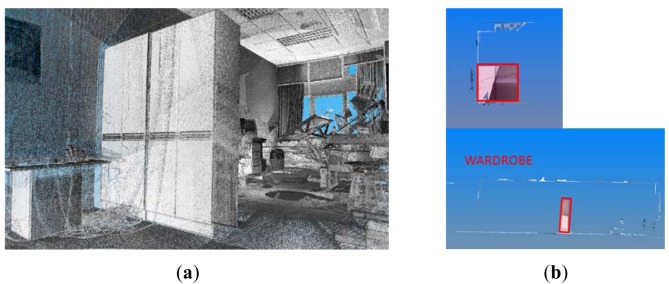
(**a**) Cloud of points including different wardrobes models inserted in the pose calculated. (**b**) Front and lateral views of a segmented wardrobe.

Wardrobes and cabinets tend to be placed against a wall and are parallelepipeds, as shown in Figure 8. The height of their tops is usually higher than the laser scanner's height, and it is therefore very probable that the top faces are not sensed. Nevertheless, the sensor will capture points in at least two vertical adjacent orthogonal planes (except in the case of several adjacent cupboards). A procedure with which to search for vertical polygons of known dimensions in the 3D data remaining from the previous stages is then carried out. Boundaries are established in the intersection of planes so that the support points on the floor can be roughly determined. As in the previous case, an ICP algorithm between 3D data and the nodes of the closet's 3D mesh model is then used. This is initialized putting into correspondence both the centroid of the 3D mesh model with the centroid of the set of the vertical polygons identified and the normal directions of the faces of the sensed wardrobe with the model ones.

**Figure 9.**
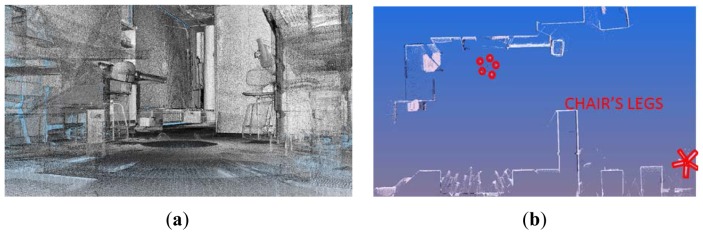
(**a**) Viewport near the floor from which several chair legs can be seen. (**b**) Data slice showing the recognized legs' patterns.

As regards the chairs, the data gathered by the RFID sensor provide information about the separation between the legs. The pattern of the chair legs is searched in a slice of points near to the floor. As in the previous case, the slice of points is transformed into an image in which 2D matching algorithms can be applied. The pattern to be matched in the image consists of a group of 3, 4 or 5 dots, or of a starry form of 3, 4 or 5 arms. These patterns can be easily identified by using cross correlation algorithms [46], matching the image with the models stored in our database. Figure 9 shows an example in which two types of chair patterns have been identified and marked in the scene. The accurate 3D position in the scene model of a detected chair pattern is again computed with an ICP algorithm. Owing to possible ambiguities in the correspondence of points, a fast test based on a least squares method is carried out to discard those which are erroneous.

## 6. Experimental Results

This section describes some of the experimental results obtained after testing our approach on a panoramic range of data appertaining to interiors of buildings in which we placed a set of RFID tags. Note that we are dealing with very complex scenarios with a high degree of clutter and occlusion. They contain a wide variety of objects that occlude not only the walls but also the ceiling and floor. In spite of occlusion problems, we want to collect as much data of the scene as possible. So, we need to take several scans per room.

Under our work conditions, the FARO Photon laser scanner provided between 30 to 40 million of points per room, depending on the number of scans. In this work we have used a set of 17 RFID tags, which are read by a LRU 3500 FEIG reader. The tags were placed from 40 cm to 600 cm from the tag reader. The main purpose of the experiences carried out in this setup was to perform reading processes on the tags and to combine this information with the laser scanner information with the aim of attaining a basic geometric and semantic model of the inhabited interior. In all the cases the RFID reader worked properly.

**Figure 10.**
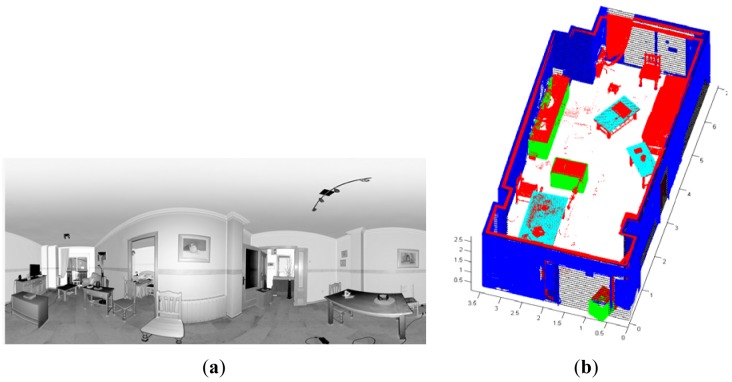
(**a**) Panoramic image of a room under study. (**b**) Walls and dynamic elements identification and positioning.

Our approach has been tested in four different inhabited interiors in which we have changed the distribution of the dynamic elements in the room aiming to test several configurations. Figure 10 shows the results for a room in which four scans were taken. In Figure 10(a) we can appreciate that some pictures and papers are adhered to walls and windows and the tables have some clutter on them. Figure 10(b) shows the results for the identification and positioning of the walls and several pieces of furniture. Three different wardrobes and three tables are detected in the scene.

**Figure 11.**
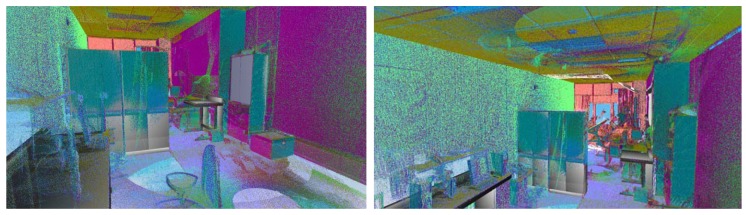
Insertion of the modeled furniture overlapped onto the cloud of points.

Figure 11 shows two views of another room in which we tested our approach. In this case, we have depicted the data provided by the sensor from four positions overlapped onto the models of the elements that the algorithm was able to recognize and pose. Owing to the presence of dynamic elements in the scene, important occlusions can be generated. This fact justifies that several scans are necessary to achieve a complete point cloud of the room. Note that, for this room, the three big wardrobes of a height of 2 m were detected in the scene. Three tables were also recognized. Two of them had tops of around 3 m^2^ and the other table, shown on the right-hand side of the figure, was much smaller. Three out of five chairs were recognized, two of which were on the front side of the room. Unfortunately two chairs were not detected because of the lack of data on their legs. Other existing objects in the scene, such as boxes on the floor, curtains, books, lights or a vertical panel were classified as clutter and were not of course recognized. Openings consisted of a double door and a large window. In both cases, the algorithm was able to detect and refine the location and size of the openings with the help of the corresponding RFID tags.

Table 2 shows the processing time in the main processes of this work. We basically give times corresponding to data acquisition, segmentation and positioning of the elements which constitute the scene. Most of the values correspond to individual processes. Thus, data acquisition time (3D and RFID) is for one position of the laser scanner and the time of 3D data processing corresponds to the registration of two different scans. The time concerning wall segmentation is for all walls, ceiling and floor. Finally, the identification and positioning time is for each piece of furniture. Note that the processes of 3D data acquisition and preprocessing are by far much more time consuming than the others.

**Figure 12.**
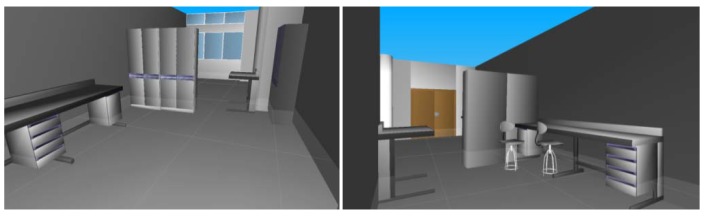
The final 3D basic model obtained after running our method.

Figure 12 illustrates the basic model of a test-room obtained after all particular recognition and pose sub-processes had been carried out. Lights and shades have been introduced in the scene in order to provide a more realistic 3D model. Note also that although the scanning of the room was carried out with an open double-door (in order to facilitate the detection), the 3D model shows the door closed.

As regards the equipment cost, the scanner is by far the most expensive component. An accurate and reliable large 3D scanner can be acquired by tens of thousand dollars whereas the RFID system costs a few thousand dollars. On the other hand, the computational time mainly depends on the data density that the scanner takes. In this case, the scanner worked with medium densities, getting one point per 0.08° × 0.08°, which provides 5.5 million points per scan.

## 7. Conclusions

To date, various partial solutions for generating automatic 3D object models using a laser scanner have been published in literature, most of them in simple and non-panoramic scenarios. In building and facility environments the obtaining of automatic 3D models becomes much more difficult because of the huge amount of information to be processed and the presence of significant clutter and occlusion, which frequently occurs in natural indoor environments.

This paper aims to fuse a laser scanner with RFID technologies, with the intention of greatly alleviating the segmentation, recognition and positioning of basic elements in an inhabited interior. As is well known, the segmentation/identification of objects among millions of points generated by the scanner is a very difficult task. RFID tags adhered to different objects can provide valuable information which facilitates the identification and positioning of basic elements of the scene. In summary, this makes the automatic modeling more reliable, faster and simpler.

The approach proposed in this paper is capable of identifying and modeling the main static structural components of an indoor environment, such as walls, floor, ceiling, windows, and doorway, along with the so-called moving or dynamic basic elements, such as tables, chairs and wardrobes/cabinets. The main idea is to refine the results provided by pure 3D data processing algorithms with the RFID tag information or to run specific algorithms for each particular object in the scene. With this strategy, we were able to provide 3D basic semantic indoor models using a FARO Photon scanner and a FEIG OBID scan UHF sensor as an RFID system.

Future improvements to the method will be addressed along various lines. Firstly, in order to improve the efficiency of the algorithms and make them more robust, a more extended test should be carried out in other scenarios. Our approach worked very well for basic planar surface detection and modeling, despite the high levels of occlusion and missing data. However, more complex room geometries and curved/free-form objects in the scene are not yet detected. New detection and segmentation approaches must therefore be investigated in the future.

Another interesting idea concerns the use of reflectance images and color information to make the object identification more robust. Finally, we are also considering improving the semantics of the reconstructed model by explicitly classifying a more extended variety of static and dynamic objects.

## Figures and Tables

**Table 1.** Summary of tag localization, and the data stored in each of them.

**Type of element**	**Tag**	**Data Stored**
	Wall	type (wall, ceiling, floor),dimension, paint color and number of openings
Static	Opening	type (door, window, closet, pure hole), dimension and color
	Molding	type (door, window), main dimensions, color
	Column	cylinder/square, main dimensions
	Table	high, area of table top, color
Dynamic	Wardrobe/Filling cabinet	dimension of three planes, color
	Chair	type( chair, sofa), chair's legs length and separation, color, surface and volume

**Table 2.** Computational time for the main processes of our proposal.

**Process**	**Time [s]**
3D Data acquisition (∼5 million points)	∼3,600
3D Data preprocessing	∼1,800
RFID Data Acquisition	<1
RFID Data Processing	<1
Wall identification and segmentation	31.57
Tables segmentation	2.93
Table identification and positioning (ICP)	1.63
Wardrobes segmentation	5.91
Wardrobe identification and positioning (ICP)	21.41
Chairs preprocessing data	99.60
Chair identification and positioning (cross correlation)	3.51
